# Medical Therapy, Radiofrequency Ablation, or Cryoballoon Ablation as First-Line Treatment for Paroxysmal Atrial Fibrillation: Interpreting Efficacy Through the Shiny Method

**DOI:** 10.7759/cureus.22645

**Published:** 2022-02-27

**Authors:** Sabrina Trippoli, Lorenzo Di Spazio, Marco Chiumente, Andrea Messori

**Affiliations:** 1 Health Technology Assessment Unit, Regione Toscana, Firenze, ITA; 2 Hospital Pharmacy Department, Santa Chiara Trento Hospital, Trento, ITA; 3 Scientific Direction, Società Italiana di Farmacia Clinica e Terapia (SIFaCT), Milano, ITA

**Keywords:** paroxysmal atrial fibrillation, first-line treatment, cryoballoon ablation, radiofrequency ablation, medical therapy

## Abstract

In patients with paroxysmal atrial fibrillation, cryoballoon ablation (CBA) and radiofrequency ablation (RFA) represent two therapeutic approaches supported by increasing literature. While both these ablation techniques play a role during different stages of the patient’s therapeutic pathway, their use as first-line is being increasingly recognized. This scoping review comparatively examined the evidence of effectiveness for these two ablation techniques.

Our analysis was limited to the evaluation of the end-point of time to recurrence of atrial fibrillation (or other forms of atrial arrhythmias), which was the primary end-point in most clinical trials. The method used for pooling the information from clinical trials (Shiny method) is original and based on an artificial intelligence (AI) method that reconstructs individual patient data from published Kaplan-Meier time-to-event curves. Because a network meta-analysis has been published on this same clinical material, one objective of the present work was to compare the meta-analytic results with those generated by the Shiny method.

A standard literature search was conducted on PubMed/Medline. Only randomized studies comparing CBA versus medical therapy, RFA versus medical therapy, or CBA versus RFA in previously untreated patients were eligible. Trials presenting a Kaplan-Meier curve to present the above-mentioned end-point were included. Patient-level data were reconstructed by application of the Shiny method. These individual patient data were then analyzed by standard statistical testing based on hazard ratio (HR) for risk of recurrence and medians of time to recurrence. Our analysis compared the two ablation treatments and medical therapy.

A total of five trials were identified through our literature search. Information from these trials was pooled according to the three treatments (CBA: three trials, n = 365; RFA: two trials, n = 99; medical therapy: five trials, n = 457). CBA showed higher effectiveness than medical therapy (HR, 0.51; 95% confidence interval (CI): 0.38 to 0.67). In comparison with medical therapy, RFA showed a numerical trend that remained far from statistical significance (HR, 0.89; 95% CI: 0.62 to 1.27). Medians for time to recurrence were 14.1 months (95% CI: 10.0 to not reached) for RFA and 11.5 months (95% CI: 9.3 to 25.3) for medical therapy. This parameter was not reached for CBA.

The current evidence from five randomized trials suggests that CBA ranks first in effectiveness, followed by RFA and medical therapy. In our comparison between the results generated by the Shiny method with those published in the previous meta-analysis, the Shiny method confirmed its ability to account for the length of follow-up in individual trials, whereas the meta-analytic approach confirmed its ability to account for the effects of randomizations performed in the trials.

## Introduction and background

In patients with paroxysmal atrial fibrillation, cryoballon ablation (CBA) and radiofrequency ablation (RFA) represent two therapeutic approaches supported by growing literature [1-6]. While both of these ablation techniques play a role during different stages of the patient’s therapeutic pathway, their use as first-line is being increasingly recognized. This scoping review aimed to comparatively examine the evidence on the effectiveness of these two ablation techniques. Our analysis was limited to the evaluation of the end-point of time to recurrence of atrial fibrillation, which was the most frequent primary end-point of included trials. The method used for pooling the information from clinical trials is original and based on an artificial intelligence method (the Shiny method) that reconstructs individual patient data from published Kaplan-Meier time-to-event curves and generates “reconstructed” patient databases.

The Shiny method [7] was published in the second half of 2021 by three researchers of the MD Anderson Center in Houston (Texas). In brief, this method analyzes the graphs of Kaplan-Meier survival curves using an automated procedure that reconstructs individual patient data from these graphs and from basic information published in the original articles of the trials. After these reconstructed databases are created, the Shiny method permits the comparison of treatments under examination through an indirect design by application of commonly used statistical tests [8-12]. Apart from the complexity of the artificial intelligence technique, one advantage of the Shiny method is that these retrospective comparisons between previously published survival curves are based on a simple operational approach. Furthermore, the graphical nature of these pooled Kaplan-Meier curves is advantageous in terms of ease of communication.

In the present study, we applied the Shiny approach based on patient data reconstruction to analyze the clinical evidence published for CBA, RFA, and medical therapy in patients with paroxysmal atrial fibrillation. We also compared the results generated by the Shiny method with those published in the previous network meta-analysis [13] focused on the same clinical material.

## Review

Methodology

Our scoping review consisted of four main phases: (a) literature search; (b) reconstruction of individual patient data; (c) statistical analysis of reconstructed survival curves; and (d) interpretation of survival data.

Literature search

We performed a literature search to identify the randomized controlled trials (RCTs) eligible for our analysis. This search was conducted on PubMed (last query on February 12, 2022) and covered the period from January 2010 to the present date. A combined search term, namely, “atrial AND fibrillation AND (radiofrequency OR cryo*),” was employed along with the filter “randomized controlled trials.” The pathway of trial selection was handled according to the Preferred Reporting Items for Systemic Review and Meta-Analyses (PRISMA) approach [14]. We also searched the Cochrane Library for any recent systematic reviews on the subject, the ClinicalTrials.gov database, and the websites of the European Medicines Agency (EMA) and the U.S. Food and Drug Administration (FDA). The above keywords were also employed for these additional searches.

**Figure 1:**
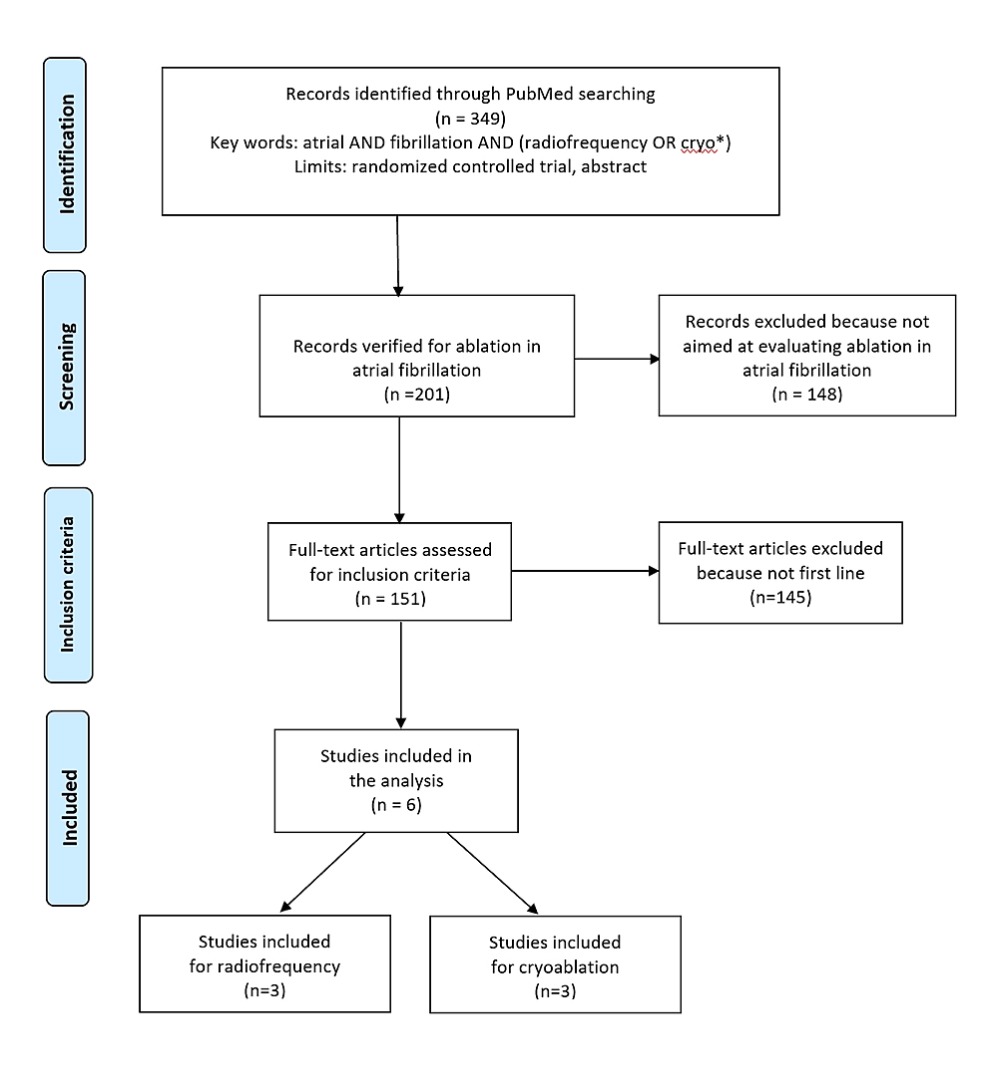
PRISMA flow diagram of our literature search. PRISMA: Preferred Reporting Items for Systemic Review and Meta-Analyses

Our analysis included the trials that met the following criteria: (a) previously untreated patients with paroxysmal atrial fibrillation; (b) randomized design; (c) treatment arm receiving either CBA or RFA; (d) control arm receiving medical therapy as first-line strategy; and (d) time-to-event end-point (freedom from recurrence) reported as a Kaplan-Meier curve (where recurrence could be either atrial fibrillation or another atrial arrhythmia). Trials with no medical therapy arm could be included provided that the two arms under comparison consisted of CBA and RFA. For each trial, we extracted the basic information needed for our analysis; information on the disease condition at baseline was recorded as well. Our literature search is summarized in Figure 1 according to the PRISMA schematic.

Application of the Shiny method and statistical analysis of reconstructed curves

In the analysis of each treatment arm of each trial, first, we reconstructed patient-level data from the Kaplan-Meier curve using the Shiny method [7]. For this purpose, each of the 10 Kaplan-Meier curves was first digitized using Webplotdigitizer (version 4.5 online; https://apps.automeris.io/wpd/). It should be noted that the two curves published by Morillo et al. [3] were ascending curves (where y = cumulative probability of event occurrence), and so these two curves were converted into descending curves (where y = cumulative probability of event-free survival). Finally, the 10 recurrence-free Kaplan-Meier curves were input into the “Reconstruct individual patient data from Kaplan-Meier survival curve” subroutine of the Shiny software (version: 1.2.2.0 online; last update: April 1, 2021); the total number of patients and the total number of events were input as well. Application of this subroutine generated 10 sets of individual patient data. Finally, standard survival statistics (Cox statistics for time-to-event end-points; package “survival” under the R-platform [15]) was performed. The hazard ratio (HR) with 95% confidence interval (CI) was the parameter employed in these analyses. Medians of recurrence-free time (with 95% CI) were also determined.

Included clinical trials

Our literature search extracted a total of 30 eligible papers. Among these, six RCTs were eligible for our analysis. After one of these six RCTs [6] was found to not report a Kaplan-Meier curve, we included five trials in our analysis [1-5]. The PRISMA schematic is reported in Figure 1. Table 1 summarizes some basic information about the five included trials.

**Table 1:** Characteristics of the ten cohorts studied in the five included trials.

Dataset	Cohort	Number of patients	Number of events	
Andrade et al. 2021 [1]	Cryoballoon	154	66	
	Medical therapy	149	101	
Wazni et al. 2021 [2]	Cryoballoon	104	21	
	Medical therapy	99	35	
Morillo et al. 2014 [3]	Radiofrequency	66	36	
	Medical therapy	61	44	
Wazni et al. 2005 [4]	Radiofrequency	33	4	
	Medical therapy	37	22	
Kuniss et al. 2021 [5]	Cryoballon	107	15	
	Medical therapy	111	33	

Generation of reconstructed time-to-event curves

**Figure 2:**
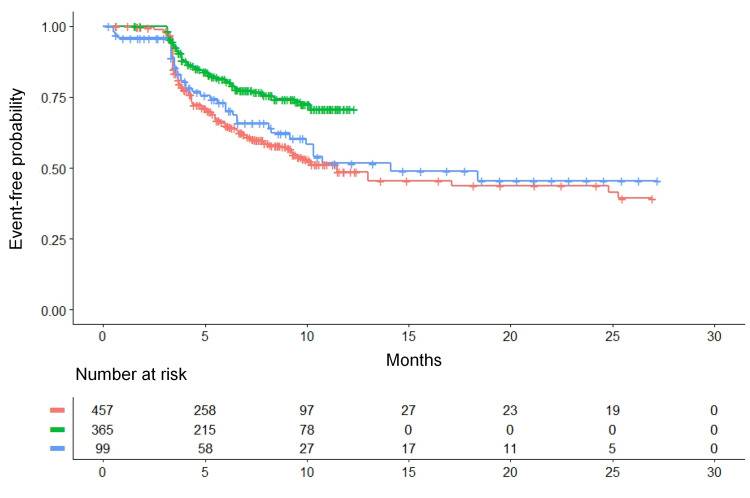
Kaplan-Meier time-to-event curves generated from reconstructed patient-level data. The three curves refer to cryoballon ablation, radiofrequency ablation, and medical therapy. Recurrence of atrial fibrillation or of another atrial arrhythmia is the time-to-event end-point. The clinical material for this analysis is reported in Table 1 (see text for further details). Identification of the curves: in green, cryoballon ablation (three trials, n = 365); in blue, radiofrequency ablation (two trials, n = 99); in red, medical therapy (five trials, n = 457). n is the number of patients.

Our analysis generated the three Kaplan-Meier curves (Figure 2) that refer to CBA, RFA, and medical therapy. In a separate analysis, we also investigated to what extent the five patient groups receiving medical therapy had homogeneous event-free patterns.

Statistical analysis of time-to-event curves

The results of our re-analysis based on reconstructed patient data (Figure 2) were the following: Medians of recurrence-free survival: (a) CBA (n = 365): not reached; (b) RFA (n = 99): 14.1 months (95% CI, 10.0 to not reached); (c) medical therapy (n = 457): 11.5 months (95% CI, 9.31 to 25.3). Pairwise comparisons versus medical therapy: (a) CBA versus medical therapy: HR = 0.51 (95% CI, 0.38 to 0.67; p < 0.001); (b) RFA versus medical therapy: HR = 0.89 (95% CI, 0.62 to 1.27; p = 0.52). Pairwise comparisons between ablation treatments: (a) CBA versus RFA: HR = 0.57 (95% CI, 0.36 to 0.91; p < 0.05).

According to these results, CBA yielded a significant improvement in event-free survival compared with the controls. RFA fared slightly better than controls but not at levels of statistical significance. In comparison with medical therapy, the improvement estimated according to the HR was greater for CBA than for RFA. The indirect comparison between CBA versus RFA favored the former at levels of statistical significance.

**Figure 3:**
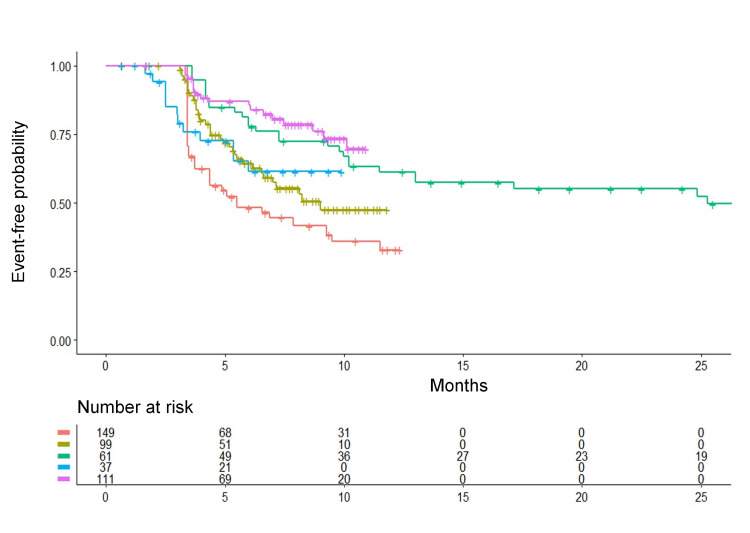
Kaplan-Meier time-to-event curves generated from reconstructed patient-level data. The curves refer to the five control groups receiving medical therapy in the five randomized trials. Relapse of atrial fibrillation or of another atrial arrhythmia is the time-to-event end-point (see text for further details). Identification of the control groups of the five trials: in red, Andrade et al. (2021) [1] (n = 149); in dark green, Wazni et al. (2021) [2] (n = 99); in light green, Morillo et al. (2014) [3] (n = 61); in blue, Wazni et al. (2005) [4] (n = 37); in purple, Kuniss et al. (2021) [5] (n = 111). n is the number of patients.

In the separate analysis that evaluated the degree of homogeneity across the five control groups receiving medical therapy, our results showed remarkable between-study differences (Figure 3). Using the control group of the trial by Andrade et al. [1] as a common comparator, the remaining four control groups from the other trials showed a better recurrence-free survival pattern. The values of HR were the following: trial by Wazni et al. (2021) [2]: HR = 0.65 (95% CI, 0.43 to 0.96; p = 0.031); trial by Morillo et al. (2014) [3]: HR = 0.38 (95% CI, 0.24 to 0.61; p < 0.001); trial by Wazni et al. (2005) [4]: HR = 0.60 (95% CI, 0.33 to 1.10; p = 0.10); trial by Kuniss et al. (2021) [5]: HR = 0.29 (95% CI, 0.17 to 0.47; p < 0.001). As shown by these results, most pair-wise comparisons between individual control groups demonstrated a significant difference. Interestingly enough, this heterogeneity in the clinical material of the five control groups had not clearly emerged in the previous analysis [13] based on restricted mean survival time (RMST). It should be kept in mind that, when individual patient data are concerned, the datasets are unsuitable for being analyzed by traditional methods of heterogeneity analysis typical of binary meta-analysis (e.g. I^2^).

Discussion

The analysis presented in this paper can be of interest, especially because a new method (the Shiny method) has been used for survival analysis. The main feature of the Shiny method is that individual patient data are automatically reconstructed from Kaplan-Meier curves published in the original trials. To compare treatments with one another, a pooled Kaplan-Meier graph is generated that includes all reconstructed patients.

The Shiny method, which is freely available on the Internet, is specifically designed to handle time-to-event end-points, which represents an area in which methodological recommendations for conducting meta-analyses have not yet been recognized, and the development of further methods of analysis is still worthwhile.

In general, the main critical aspect in the application of standard meta-analytic techniques to time-to-event end-points is represented by the inability to account for the different follow-up lengths across included studies. In most cases, this problem is solved empirically by ignoring the follow-up length and expressing the results according to the typical parameters of binary meta-analysis that disregard follow-up (e.g., relative risk or odds ratio). In the field of catheter ablation techniques for patients with atrial fibrillation, the choice to use this simplified, empirical approach finds confirmation in the two recent meta-analyses published by Imberti et al. [16] and by Kheiri and Nazer [17] that have expressed their results according to relative risk or risk ratio and have ignored the length of the follow-up. In the case of this dataset, most clinical benefits resulting from the different treatments occurred from zero to twelve months, and so the ability of the Shiny method to account for the different follow-up lengths had a negligible impact. However, this advantage of the Shiny method may be particularly important with other datasets in which the lengths of follow-up differ markedly across studies. As regards the RMST (another methodological tool available in this area [13]), its main problem consists in the poor acceptance by the scientific community.

Comparing the results provided by the Shiny method with those published in the previous RSMT-based meta-analysis [13] offers some interesting clues about the advantages and disadvantages of these two methods. One advantage of the meta-analysis is that direct comparisons can be weighted more than indirect comparisons, whereas the Shiny method weights them equally. In fact, a standard binary meta-analysis strongly characterizes each randomized trial on the basis of its own control group, whereas the Shiny method does not. Hence, if one considers the same effectiveness found in a given treatment group of a trial, a binary meta-analysis weights this trial to a lesser extent if the controls have fared better and to a greater extent if the controls have fared worse. This advantageous feature of meta-analyses depends on the link with randomization that a meta-analysis retains whereas the Shiny method does not. On the other hand, as pointed out above, one important disadvantage of meta-analysis is that the length of follow-up is not accounted for. 

Interpreting the heterogeneity found among the control groups of the five trials (Figure 3) is not straightforward. In the previous paper [13], the network meta-analysis assigned more weight to the association of each treatment group with its respective control group, which correctly reflected the effects of randomization. On the other hand, in cases where the control groups show a clear heterogeneity despite similar inclusion criteria, the approach implemented by the Shiny method (in which the different control groups are pooled into a single patient group) is advantageous because the representativeness of this pooled control group is increased. As regards the present dataset, the control groups of CBA trials fared better than the control groups of RFA trials, which explains why CBA ranked first according to the Shiny method whereas RFA ranked first in the meta-analysis. Interestingly enough, in the recent binary meta-analysis published by Imberti et al [16], both CBA and RFA significantly improved recurrence-free survival in comparison with medical therapy; according to this end-point, CBA was found to be slightly more effective than RFA, but the difference remained far from statistical significance. The binary meta-analysis of Imberti et al. was based on the relative risk; events were collected over the entire length of the different follow-ups, but no adjustment was made for these different lengths.

Overall, the solution to these issues of adjustment for different control groups and for different lengths in their follow-up may depend mainly on the clinical side of the problem, and particular attention should probably be given to carefully examining the inclusion criteria across different trials along with the potential effects of these differences. For example, in the present analysis, the differences in the clinical material between CBA and RFA have likely played an important role. Factors that can be considered as the main sources of heterogeneity include different drugs used in the medical therapy arms (the proportion of g. class III drugs), different acute success rates, and different arrhythmia recurrence detection (e.g., loop-recorder vs seven-day Holter). Another factor influencing outcomes in the controls is the likely improvement in the management of these patients that occurred between 2005 and 2021.

One important strength of the Shiny approach is related to the graphical format in which the results are presented. This format, where the analysis is synthesized through a single multi-treatment Kaplan-Meier graph, appears to be particularly attractive in terms of communication. Other advantages are related to the descriptive approach adopted by the Shiny method. For example, with respect to the heterogeneity of the five control groups, the Shiny approach identified important information, as reported in Figure 3, that the previous analysis based on RMST [13] had not identified. On the other hand, the main limitation of the Shiny method is that, despite its use of individual patient data, no multivariate analyses can be done.

This scoping review has some important limitations. From an editorial point of view, some duplication between the RMST analysis, published in 2021 [13], and the present report was unavoidable. While this partial duplication has reduced the originality of this re-analysis, the undoubted peculiarities of the Shiny method are noteworthy and support the original nature of the findings reported here.

Finally, in this phase in which the Shiny method, combined with indirect comparisons, is being applied to a growing number of different therapeutic issues (with particular emphasis on innovative treatments [9-12]), the main questions addressed by every new paper are two-fold: there are questions of clinical nature related to the specific clinical topic under examination, and there are questions of methodological nature related to the performance of the Shiny method in providing its synthesis of clinical evidence. As the Shiny method acquires growing acceptance by the scientific community, new papers will hopefully focus more on the specific clinical problem under examination than on the methodological issues raised by the Shiny method.

## Conclusions

This paper, based on the application of the Shiny method, has presented a re-analysis of a therapeutic issue previously studied through a network meta-analysis based on the RMST. In this study, the graph with pooled Kaplan-Meier curves for CBA, RFA, and medical therapy offered an effective summary of the current clinical evidence. Despite the loss of some useful information related to the effects of randomization, the Shiny method has one remarkable advantage because it accounts for the follow-up length. This advantage can be important when the probability of experiencing the end-point is projected in the long term (e.g., in oncologic diseases and cardiovascular diseases). As regards the clinical results of our analysis, both catheter ablation techniques prove to be more effective than medical therapy. When estimated according to the Shiny method, our results in terms of clinical relevance and statistical significance are more in favor of CBA than those reported in the previous meta-analyses.

## References

[1] Andrade JG, Wells GA, Deyell MW (2021). Cryoablation or drug therapy for initial treatment of atrial fibrillation. N Engl J Med.

[2] Wazni OM, Dandamudi G, Sood N (2021). Cryoballoon ablation as initial therapy for atrial fibrillation. N Engl J Med.

[3] Morillo CA, Verma A, Connolly SJ (2014). Radiofrequency ablation vs antiarrhythmic drugs as first-line treatment of paroxysmal atrial fibrillation (RAAFT-2): a randomized trial. JAMA.

[4] Wazni OM, Marrouche NF, Martin DO (2005). Radiofrequency ablation vs antiarrhythmic drugs as first-line treatment of symptomatic atrial fibrillation: a randomized trial. JAMA.

[5] Kuniss M, Pavlovic N, Velagic V (2021). Cryoballoon ablation vs. antiarrhythmic drugs: first-line therapy for patients with paroxysmal atrial fibrillation. Europace.

[6] Cosedis Nielsen J, Johannessen A, Raatikainen P (2012). Radiofrequency ablation as initial therapy in paroxysmal atrial fibrillation. N Engl J Med.

[7] Liu N, Zhou Y, Lee JJ (2021). IPDfromKM: reconstruct individual patient data from published Kaplan-Meier survival curves. BMC Med Res Methodol.

[8] Messori A (2021). Synthetizing published evidence on survival by reconstruction of patient-level data and generation of a multi-trial Kaplan-Meier curve. Cureus.

[9] Messori A, Rivano M, Mengato D, Cancanelli L, Di Spazio L, Chiumente M (2021). A preliminary estimate of survival gain and cost-effectiveness of CAR-T in adult patients with acute lymphoblastic leukemia [In Press]. Leuk Lymphoma.

[10] Cancanelli L, Rivano M, Di Spazio L, Chiumente M, Mengato D, Messori A (2021). Efficacy of immune checkpoint inhibitors in patients with mismatch repair-deficient or microsatellite instability-high metastatic colorectal cancer: analysis of three phase-II trials. Cureus.

[11] Di Spazio L, Rivano M, Cancanelli L, Chiumente M, Mengato D, Messori A (2022). The degree of programmed death-ligand 1 (PD-L1) positivity as a determinant of outcomes in metastatic triple-negative breast cancer treated with first-line immune checkpoint inhibitors. Cureus.

[12] Messori A (2022). Current treatments for advanced or metastatic osteosarcoma: indirect comparisons based on individual patient data reconstructed retrospectively from 5 trials [In Press]. J Bone Res.

[13] Messori A, Bartoli L, Ferracane E, Trippoli S (2021). Medical therapy, radiofrequency ablation or cryoballoon ablation as first-line treatment for paroxysmal atrial fibrillation: interpreting efficacy through restricted mean survival time and network meta-analysis. Rev Cardiovasc Med.

[14] Liberati A, Altman DG, Tetzlaff J (2022). The PRISMA statement for reporting systematic reviews and meta-analyses of studies that evaluate healthcare interventions: explanation and elaboration. BMJ.

[15] (2022). R Foundation for Statistical Computing: A language and environment for statistical computing. https://www.R-project.org/.

[16] Imberti JF, Ding WY, Kotalczyk A (2021). Catheter ablation as first-line treatment for paroxysmal atrial fibrillation: a systematic review and meta-analysis. Heart.

[17] Kheiri B, Nazer B (2022). Meta-analysis of catheter ablation as first-line therapy for paroxysmal atrial fibrillation. Am J Cardiol.

